# Development and validation of a nomogram to predict spontaneous preterm birth in singleton gestation with short cervix and no history of spontaneous preterm birth

**DOI:** 10.1016/j.heliyon.2023.e20453

**Published:** 2023-09-27

**Authors:** Yongkang Sun, Feng Lian, Yuanyuan Deng, Sha Liao, Ying Wang

**Affiliations:** Department of Ultrasound, Seventh People's Hospital of Shanghai University of Traditional Chinese Medicine, Shanghai, 200137, PR China

**Keywords:** Spontaneous preterm birth, Singleton gestation, Short cervix, Nomogram

## Abstract

**Background:**

Spontaneous preterm birth (sPTB) stands as a leading cause of neonatal mortality. Consequently, preventing sPTB has emerged as a paramount concern in healthcare. Therefore, our study aimed to develop a nomogram, encompassing patient characteristics and cervical elastography, to predict sPTB in singleton pregnancies. Specifically, we targeted those with a short cervix length (CL), no history of sPTB, and who were receiving vaginal progesterone therapy.

**Methods:**

A total of 568 patients were included in this study. Data from 392 patients, collected between January 2016 and October 2019, constituted the training cohort. Meanwhile, records from 176 patients, spanning November 2019 to January 2022, formed the validation cohort. Following the univariate logistic regression analysis, variables exhibiting a P-value less than 0.05 were integrated into a multivariable logistic regression analysis. The primary objective of this subsequent analysis was to identify the independent predictors linked to sPTB in the training cohort. Next, we formulated a nomogram utilizing the identified independent predictors. This tool was designed to estimate the likelihood of sPTB in singleton pregnancies, particularly those with a short CL, devoid of any sPTB history, and undergoing vaginal progesterone therapy. The C-index, Hosmer-Lemeshow (HL) test, calibration curves, decision curve analysis (DCA), and receiver operating characteristic (ROC) were used to validate the performance of the nomogram.

**Results:**

Upon finalizing the univariate analysis, we progressed to a multivariable analysis, integrating 8 variables with P < 0.05 from the univariate analysis. The multivariable analysis identified 7 independent risk factors: maternal age (OR = 1.072; P < 0.001), cervical length (OR = 0.854; P < 0.001), uterine curettage (OR = 7.208; P < 0.001), GDM (OR = 3.570; P = 0.006), HDP (OR = 4.661; P = 0.003), C-reactive protein (OR = 1.138; P < 0.001), and strain of AI (OR = 7.985; P < 0.001). The nomogram, tailored for sPTB prediction, was grounded on these 7 independent predictors. In predicting sPTB, the C-indices manifested as 0.873 (95% CI, 0.827–0.918) for the training cohort and 0.916 (95%CI, 0.870–0.962) for the validation cohorts, underscoring a good discrimination of the model. Additionally, the ROC curves served to evaluate the discrimination of nomogram model across both cohorts. Calibration curves were delineated, revealing no statistically significant differences in both the training (χ^2^ = 5.355; *P* = 0.719) and validation (χ^2^ = 2.708; *P* = 0.951) cohorts as evidenced by the HL tests. Furthermore, the DCA underscored the model's excellence as a predictive tool for sPTB.

**Conclusions:**

By amalgamating patient characteristics and cervical elastography data from the second trimester, the nomogram emerged as a visually intuitive and dependable tool for predicting sPTB. Its relevance was particularly pronounced for singleton pregnancies characterized by a short CL, an absence of prior sPTB incidents, and those receiving vaginal progesterone therapy.

## 1 Introduction

Globally, nearly 15 million spontaneous preterm births (sPTBs), which are births occurring before 37 weeks of gestation, are reported annually. Alarmingly, this incidence is on the rise in some countries [[1], [2], [3]]. A staggering 12 million of these sPTBs are concentrated in Africa and Asia, making up the vast majority of the global total [4]. Complications arising from sPTBs, such as cerebral palsy, respiratory issues, and intellectual disabilities, significantly contribute to neonatal mortality. Consequently, preventing sPTBs has become a paramount healthcare concern [5]. A notable risk factor for sPTB is a previous occurrence; women who have experienced an sPTB in the past face an approximately 20% risk of recurrence [6]. However, it's worth noting that, due to the limited number of women with a history of sPTB, most women who experience sPTB do not have a prior record of such births [7]. Furthermore, during the second trimester (20–24 weeks), women with short cervix length (CL) are at a heightened risk of sPTB [[8], [9], [10]]. While a normal CL is typically ≥25 mm, any measurement below this is categorized as short [9,11,12]. Studies have shown that vaginal progesterone can significantly decrease the risk of sPTB in singleton pregnancies with a short CL during this period, especially when compared to a placebo [13]. However, even with the administration of vaginal progesterone, the reduction in sPTB cases was modest, with the rate still exceeding 11% [[14], [15], [16]]. As a result, there's a growing emphasis on identifying predictors of sPTB in women who have a short CL, no prior sPTB, and are undergoing vaginal progesterone treatment. The primary objective of this focus is to facilitate timely interventions and subsequently lower the occurrence of sPTB.

Certain patient characteristics, including advanced maternal age, being overweight or underweight, undergoing multiple uterine curettages, and utilizing assisted reproductive technologies (ART), play a significant role in the onset of sPTB [17,18]. Furthermore, the premature softening of the cervix is linked to sPTB. Evaluating changes in cervical stiffness through cervical elastography has proven beneficial in predicting sPTB [19]. While numerous studies have validated the efficacy of cervical elastography in this regard [[20], [21], [22], [23]], few have explored its combined use with patient characteristics, especially in women with a short CL, no prior of sPTB history, and those undergoing vaginal progesterone treatment.

The aim of our study was to develop a nomogram that integrated both patient characteristics and cervical elastography findings. This tool aimed to predict sPTB in singleton pregnancies, especially in cases with a short CL, no prior of sPTB history, and those undergoing vaginal progesterone treatment. Through our study, we offer clinicians a valuable instrument to implement preventive strategies and mitigate the adverse outcomes associated with sPTB.

## 2 Materials and methods

The institutional review board of Seventh People's Hospital of Shanghai University of Traditional Chinese Medicine approved this study (No. IEC-C-014-V1.0). The informed consent was obtained from all participants in written or oral form by telephone, email, WeChat, and others. It was conducted in compliance with the Declaration of Helsinki.

### 2.1 Study participants

**Fig. 1:**
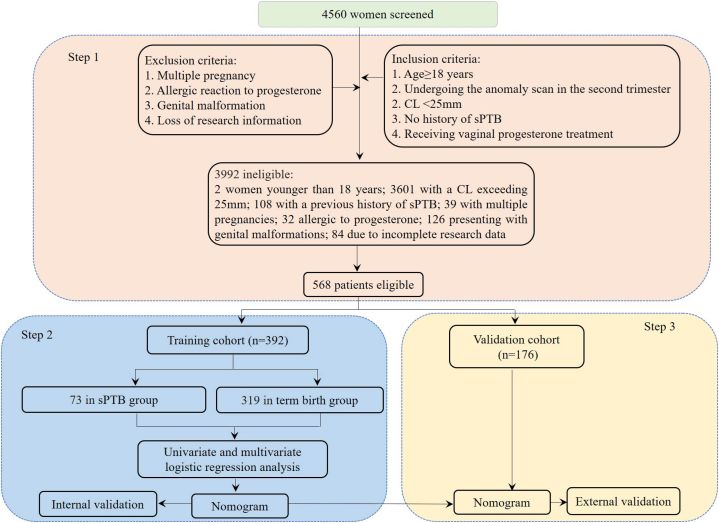
Summary of patient flow diagram. Step 1: patient screening; Step 2: model construction; Step 3: model validation; sPTB, spontaneous preterm birth; CL, cervix length.

Inclusion criteria included: age ≥18 years; undergoing an anomaly scan during the second trimester (20–24 weeks); a CL measurement of less than 25 mm; no prior history of sPTB; and administration of vaginal progesterone treatment from 20 to 24 to 34–36 weeks of gestation. The exclusion criteria included: multiple pregnancies; known allergies to progesterone; genital malformation; or missing research data. From January 2016 to January 2022, we screened of 4560 women at Seventh People's Hospital of Shanghai University of Traditional Chinese Medicine. Among then, the following were excluded: 2 women younger than 18 years, 3601 with a CL exceeding 25 mm, 108 with a previous history of sPTB, 39 with multiple pregnancies, 32 allergic to progesterone, 126 presenting with genital malformations, and 84 due to incomplete research data. Consequently, a total of 568 patients were enrolled in this study. To mitigate the risk of overfitting, we partitioned the enrolled patients into training and validation cohorts at an approximate ratio of 7:3, organized in chronological order based on their diagnosis dates. Data from January 2016 to October 2019, comprising 392 patients, constituted the training cohort. This cohort was further categorized into subgroups: the sPTB group and term birth group. Meanwhile, data from November 2019 to January 2022, which included 176 patients, was designated as the validation cohort (Fig. 1). Adhering to the rule that recommends at least 10 outcome events per variable [24,25], we ensured the model retained fewer than 10 features to further prevent overfitting.

### 2.2 CL and elastographic measurements

The transvaginal ultrasound examination was carried out using Philips IU Elite Ultrasound System, equipped with a transvaginal probe, to measure the CL and conduct cervical elastography. The probe was carefully inserted into the anterior fornix of the vagina to achieve a sagittal view of the cervix. The CL was identified as the distance between the internal and external cervical os, ensuring no pressure was applied to the cervix during the measurement.

Elastography was conducted by applying external pressure to the anterior lip of the cervix, using gentle and rhythmic movements. The procedure utilized a green elastic scale specifically designed for strain image acquisition. This scale, displaying a strain range from 0 to 1, transitioned in color from blue to red, indicative of tissue stiffness to softness, respectively. This aided in calculating tissue deformation. Four specific regions of interest (ROIs) were identified on the cervix: the anterior lip of the internal os (AI), the posterior lip of the internal os (PI), the anterior lip of the external os (AE), and the posterior lip of the external os (PE). The average strain was then determined for each of these ROIs.

### 2.3 Data collection

This study recorded data encompassing various patient characteristics, including maternal age, pre-pregnancy BMI, CL during the second trimester, parity, uterine curettage, and histories of alcohol consumption, smoking, diabetes mellitus in the family, hypertension, gestational diabetes mellitus (GDM), and hypertensive disorders of pregnancy (HDP). Other factors considered were anemia (defined by haemoglobin concentration <110 g/L), C-reactive protein levels, urinary and reproductive tract infections, inter-pregnancy interval, gestational age at birth, annual household income, emergency cerclage, ART, and antibiotics usage. Additionally, the average strain of AI, PI, AE, and PE in the cervix was documented. Patients' haemoglobin and C-reactive protein levels were assessed during their initial outpatient department visit. GDM was characterized by the presence of at least 1 plasma glucose anomaly (fasting plasma glucose≥5.1 mmol/L, 60-min ≥10.0 mmol/L, 120-min ≥8.5 mmol/L by 75 g oral glucose tolerance test [OGTT]) during the second trimester of pregnancy. HDP encompassed conditions like pregnancy-induced hypertension and preeclampsia.

### 2.4 Nomogram construction

Analyses of risk factors were conducted using both univariate analysis and multivariate logistic regression model, employing the backward stepwise method. Upon completing the univariate logistic regression analysis, we proceeded with a multivariable logistic regression analysis, incorporating variables with a significance level of P < 0.05 from the univariate analysis. This multivariable analysis pinpointed the independent risk factors linked to sPTB within the training cohort. Using these independent predictors, we developed a nomogram to estimate the sPTB risk in singleton pregnancies characterized by a short CL, absence of sPTB history, and undergoing vaginal progesterone treatment. We determined the nomogram coefficients for each variable based on the formula: Nomogrami = (max (Wi) − min (Wi) ∗ βi, where max (W) and min (W) denote the maximum and minimum values of each respective variable. Next, we chose strain of AI as the scoring scale, setting a total score of 100. After establishing the score aligned with the strain of AI, we calculated the score for each variable using the coefficient: Pointi = 100 ∗ (Nomogrami/NomogramAI)/(max (Wi) −min (Wi)).

### 2.5 Validation of the nomogram

To validate the nomogram, we employed bootstrap resampling (1000 times) analysis in both the training and validation cohorts. The calibration ability of this nomogram was assessed by the calibration curve and Hosmer-Lemeshow (HL) test. The receiver operating characteristic (ROC) curve and C-index were used to assess the discrimination of the nomogram. Additionally, we conducted a decision curve analysis (DCA) to determine its the clinical net benefits.

### 2.6 Statistical analysis

Of the 568 enrolled patients, 106 experienced sPTB. Adhering to the rule that suggests at least 10 outcome events per variable [24,25], we ensured that the model retained fewer than 10 features to maintain sufficient statistical power. Data with a normal distribution were analyzed using the independent sample *t*-test, while data with a skewed distribution were assessed with the Mann–Whitney *U* test. Categorical variables such as gravidity, parity, uterine curettage, and anemia were compared using the Chi-square test. After the univariate logistic regression analysis, a multivariable logistic regression model was established in the training cohort to identify the independent predictors of sPTB, aiding in the construction of nomogram. All statistical analyses were performed using the R package (version 3.6.2), with a significance level set at *P* < 0.05.

## 3 Results

Among the 4560 pregnant women screened, 568 who met the inclusion and exclusion criteria and had singleton pregnancies were selected for our study. These women underwent an anomaly scan during their second trimester (20–24 weeks), as illustrated in Fig. 1. Of these, 73 (18.6%) women from the training cohort and 33 (18.8%) from the validation cohort experienced sPTB. In the sPTB group, the rates of moderate to late preterm (32–36 weeks' gestation) were 87.7% in the training cohort and 85.8% in the validation cohort. The rates of very preterm (28–31 weeks' gestation) were 11.4% and 13.2% for the training and validation cohorts, respectively. The extremely preterm (<28 weeks’ gestation) occurrences were 0.9% for the training cohort and 1.0% for the validation cohort. Table 1 presented the patient characteristics and elastic data, indicating no significant differences between the two cohorts (all *P* values > 0.05).

**Table 1:** Baseline characteristics of patients in the training and validation cohorts.

Variable	Training Cohort (n = 392)	Validation Cohort (n = 176)	*P* value
**Patient characteristics**			
Maternal age, years, mean ± SD	33.97 ± 8.65	34.57 ± 8.49	0.441^a^
BMI before pregnancy, kg/m^2^, mean ± SD	25.18 ± 5.81	25.06 ± 5.96	0.825^a^
Cervical length, mm, mean ± SD	16.16 ± 4.88	15.67 ± 4.55	0.258^a^
Parity, n (%)			0.176^b^
0	265 (67.6)	108 (61.4)	
≥1	127 (32.4)	68 (38.6)	
Uterine curettage, n (%)			0.948^b^
Yes	40 (10.2)	19 (10.8)	
No	352 (89.8)	157 (89.2)	
Alcohol drinking history, n (%)			0.747^b^
Yes	43 (11.0)	17 (9.7)	
No	349 (89.0)	159 (90.3)	
Smoking history, n (%)			0.987^b^
Yes	5 (1.3)	3 (1.7)	
No	387 (98.7)	173 (98.3)	
Family history of diabetes mellitus, n (%)			0.943^b^
Yes	38 (9.7)	16 (9.1)	
No	354 (90.3)	160 (90.9)	
History of hypertension, n (%)			0.845^b^
Yes	14 (3.6)	5 (2.8)	
No	378 (96.4)	171 (97.2)	
GDM, n (%)			0.918^b^
Yes	44 (11.2)	21 (11.9)	
No	348 (88.8)	155 (88.1)	
HDP, n (%)			0.983^b^
Yes	33 (8.4)	14 (8.0)	
No	359 (91.6)	162 (92.0)	
Anemia, n (%)			1.000^b^
Yes	52 (13.3)	24 (13.6)	
No	340 (86.7)	152 (86.4)	
C-reactive protein, mg/L, mean ± SD	7.81 ± 4.69	8.02 ± 4.95	0.631^a^
Urinary tract infection, n (%)			0.964^b^
Yes	31 (7.9)	13 (7.4)	
No	361 (92.1)	163 (92.6)	
Reproductive tract infection, n (%)			0.917^b^
Yes	55 (14.0)	26 (14.8)	
No	337 (86.0)	150 (85.2)	
Inter-pregnancy interval, months, mean ± SD	39.34 ± 21.27	40.69 ± 24.89	0.507^a^
Household yearly income (yuan), n (%)			0.958^b^
<50,000	203 (51.8)	90 (51.1)	
≥50,000	189 (48.2)	86 (48.9)	
Emergency cerclage, n (%)			0.207^b^
Yes	12 (3.1)	10 (5.7)	
No	380 (96.9)	166 (94.3)	
Assisted reproductive technologies, n (%)			1.000^b^
Yes	22 (5.6)	10 (5.7)	
No	370 (94.4)	166 (94.3)	
Antibiotics use, n (%)			0.974^b^
Yes	87 (22.2)	40 (22.7)	
No	305 (77.8)	136 (77.3)	
**Strain in the cervix**			
AI, mean ± SD	0.27 ± 0.09	0.27 ± 0.10	0.844^a^
PI, mean ± SD	0.31 ± 0.12	0.31 ± 0.12	0.994^a^
AE, mean ± SD	0.41 ± 0.11	0.42 ± 0.12	0.197^a^
PE, mean ± SD	0.43 ± 0.11	0.45 ± 0.11	0.141^a^

a , for independent sample *t*-test.

b , for chi-square test; SD, standard deviation; BMI, body mass index; GDM, gestational diabetes mellitus; HDP, hypertensive disorders of pregnancy; AI, anterior lip of internal os; AE, anterior lip of external os; PI, posterior lip of internal os; PE, posterior lip of external os.

### 3.1 Independent predictors for sPTB

In the training cohort, univariate analysis indicated significant associations between sPTB and factors such as maternal age, cervical length, parity, uterine curettage, GDM, HDP, C-reactive protein, and strain of AI (all P < 0.05) (Table 2). These findings were further corroborated using the independent sample *t*-test, chi-square test, or Mann-Whitney *U* test (Supplementary Table 1). Following the univariate analysis, a multivariable analysis was conducted, incorporating the 8 variables with P < 0.05 from the initial analysis. This multivariable analysis identified 7 independent risk factors: maternal age (OR = 1.072; P < 0.001), cervical length (OR = 0.854; P < 0.001), uterine curettage (OR = 7.208; P < 0.001), GDM (OR = 3.570; P = 0.006), HDP (OR = 4.661; P = 0.003), C-reactive protein (OR = 1.138; P < 0.001), and strain of AI (OR = 7.985; P < 0.001) (Table 2).

**Table 2:** Univariate and multivariate logistic analyses to determine the independent predictors associated with sPTB in the training cohort.

Variable	Univariate Logistic Regression			Multivariable Logistic Regression		
	*P* value	OR	95% CI	*P* value	OR	95% CI
**Patient characteristics**						
Maternal age, years	<0.001	1.070	1.037–1.105	<0.001	1.072	1.031–1.116
BMI before pregnancy, kg/m2	0.463	0.984	0.941–1.028			
Cervical length, mm	<0.001	0.856	0.804–0.908	<0.001	0.854	0.788–0.919
Parity						
0	Reference			Reference		
≥1	0.036	1.901	1.065–3.564	0.250	1.547	0.748–3.338
Uterine curettage						
No	Reference			Reference		
Yes	<0.001	5.642	2.838–11.256	<0.001	7.208	3.025–17.772
Alcohol drinking history						
No	Reference					
Yes	0.410	1.376	0.616–2.847			
Smoking history						
No	Reference					
Yes	0.238	2.967	0.386–18.222			
Family history of diabetes mellitus						
No	Reference					
Yes	0.686	1.186	0.488–2.593			
History of hypertension						
No	Reference					
Yes	0.105	2.533	0.758–7.573			
GDM						
No	Reference			Reference		
Yes	<0.001	3.283	1.656–6.382	0.006	3.570	1.432–8.849
HDP						
No	Reference			Reference		
Yes	0.002	3.239	1.498–6.812	0.003	4.661	1.661–13.027
Anemia						
No	Reference					
Yes	0.615	1.203	0.561–2.400			
C-reactive protein, mg/L	<0.001	1.138	1.075–1.206	<0.001	1.138	1.064–1.222
Urinary tract infection						
No	Reference					
Yes	0.711	0.829	0.273–2.069			
Reproductive tract infection						
No	Reference					
Yes	0.928	0.966	0.440–1.951			
Inter-pregnancy interval, months	0.105	1.008	0.998–1.019			
Household yearly income, yuan						
<50,000	Reference					
≥50,000	0.213	1.383	0.831–2.315			
Emergency cerclage						
No	Reference					
Yes	0.567	1.476	0.321–5.094			
Assisted reproductive technologies						
No	Reference					
Yes	0.288	1.696	0.590–4.295			
Antibiotics use						
No	Reference					
Yes	0.492	0.780	0.409–1.478			
**Strain in the cervix**						
AI	<0.001	6.093^a^	2.617–18.027	<0.001	7.985^a^	1.837–47.929
PI	0.411	2.513	0.281–22.903			
AE	0.758	0.704	0.074–6.628			
PE	0.677	0.616	0.062–5.964			

a Denotes a specifc OR value, indicating that the risk increases by 0.01 unit increments; sPTB, spontaneous preterm birth; BMI, body mass index; GDM, gestational diabetes mellitus; HDP, hypertensive disorders of pregnancy; AI, anterior lip of internal os; AE, anterior lip of external os; PI, posterior lip of internal os; PE, posterior lip of external os; OR, odds ratio; CI, confidence interval.

### 3.2 Nomogram establishment

**Fig. 2:**
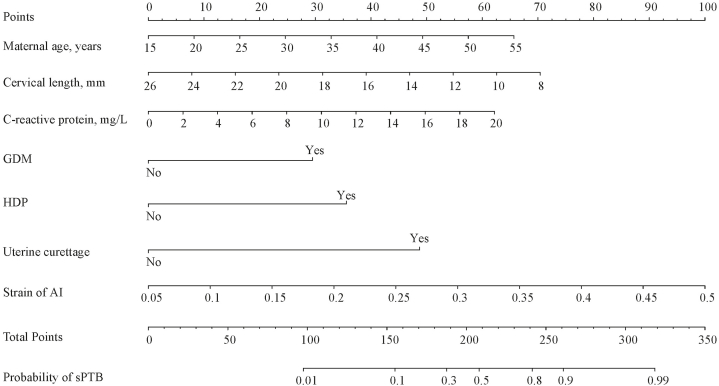
Nomogram for predictInt. J. Womens Healthing sPTB in singleton gestation with a short CL, no history of sPTB, and under vaginal progesterone treatment. GDM, gestational diabetes mellitus; HDP, hypertensive disorders of pregnancy; AI, anterior lip of internal os; sPTB, spontaneous preterm birth.

A nomogram was developed to predict sPTB, utilizing 7 independent predictors: maternal age, cervical length, uterine curettage, GDM, HDP, C-reactive protein, and strain of AI (Fig. 2). The total points, derived from the sum of these predictors, corresponded to the probability of sPTB. For instance, consider a 40-year-old woman (41 points) with a CL of 12 mm (55 points), a C-reactive protein level of 12 mg/L (37 points), a strain of AI value of 0.35 (67 points), diagnosed with GDM (29 points), without HDP (0 point), and having a history of uterine curettage (48 points). Her total score would be 277, translating to a 93% probability of experiencing sPTB.

### 3.3 Nomogram verification

**Fig. 3:**
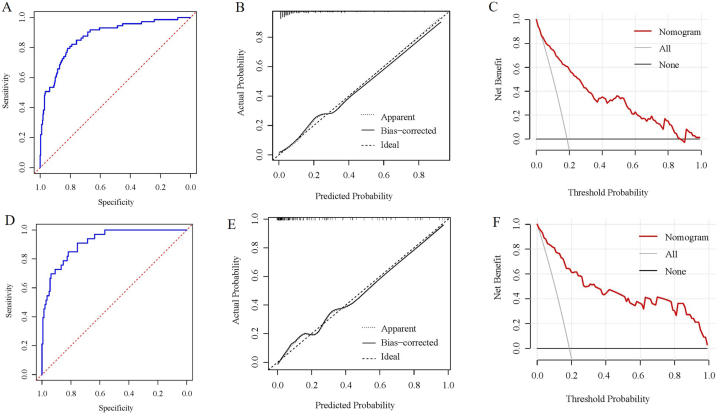
Nomogram verification (A-C for training cohort and D-F for validation cohort). (A, D) ROC curves; (B, E) calibration plots; (C, F) DCA plots. ROC, receiver operating characteristic; DCA, decision curve analysis.

The C-indices for sPTB prediction were 0.873 (95% CI, 0.827–0.918) for the training cohort and 0.916 (95%CI, 0.870–0.962) for the validation cohorts, indicating robust model discrimination. The discriminative ability of the model was further assessed using the ROC curves (Fig. 3A and D) for the respective cohorts. Calibration curves were shown in Fig. 3B and E. Notably, using the HL tests, there were no significant differences in both the training (χ^2^ = 5.355; *P* = 0.719) and validation (χ^2^ = 2.708; *P* = 0.951) cohorts. Moreover, the DCA underscored the excellence of this model as a predictive tool for sPTB (Fig. 3C and F).

## 4 Discussion

For women with a short CL, no prior history of sPTB, and undergoing vaginal progesterone treatment, accurate prediction of sPTB during their second trimester can enable more effective interventions to prevent sPTB. Addressing this need, we developed a nomogram incorporating maternal age, cervical length, uterine curettage, GDM, HDP, C-reactive protein, and strain of AI. This model aimed to predict sPTB for women fitting the aforementioned criteria between 20 and 24 weeks of gestation. Evaluations from both the training and validation cohorts showed that this nomogram model exhibited strong performance, positioning it as a potentially reliable tool for sPTB prediction.

sPTB is a major factor in neonatal mortality, making its prevention a paramount healthcare objective. While most studies have focused on the accuracy of cervical elastography in predicting of sPTB, few have suggested practical screening methods for potential sPTB using elastic data [[26], [27], [28], [29]]. Medical nomograms graphically present statistical prediction models by utilizing patient-specific variables, like age and CL. These models estimate the likelihood of specific events, such as preterm birth, for an individual. Over time, they have evolved into user-friendly tools [30,31]. Our study not only reaffirmed the strain of AI as an independent predictor for sPTB in singleton pregnancies with a short CL, no prior sPTB incidents, and undergoing vaginal progesterone treatment but also elucidated the impact of the strain of AI on sPTB via a nomogram model, aiding clinicians in assessing sPTB risks.

In our study, besides the strain of AI, factors such as maternal age, CL, uterine curettage, GDM, HDP, and C-reactive protein emerged as independent predictors of sPTB. Previous research has highlighted a strong association between advanced maternal age and sPTB [32], with Jacobsson et al. emphasizing a higher frequency of sPTB in women aged 40 years or older [33]. Evaluating CL through transvaginal ultrasound between 16 and 24 weeks of gestation has been recognized as a reliable predictor of sPTB. Notably, a shorter CL heightens the risk of sPTB, irrespective of one's reproductive history [9]. The number of uterine curettages, due to its potential to induce cervical trauma, is considered an independent predictor of sPTB [34]. It's imperative to meticulously document the number of such procedures and assess their implications on sPTB. GDM, characterized by hyperglycemia during pregnancy, has been linked to complications like sPTB [35]. With the increasing prevalence of obesity and type 2 diabetes in certain regions, the incidence of GDM is on the rise, warranting close attention [35]. HDP prevalence fluctuates between 5% and 10% [36], with endothelial dysfunction at the placental level being viewed as a potential sPTB mechanism [37]. Furthermore, Huang S et al. found a correlation between elevated C-reactive protein concentrations in the first trimester and increased sPTB risk [38]. Our findings generally aligned with existing data concerning maternal age, CL, uterine curettage, GDM, HDP, and C-reactive protein.

The main strength of this study was the development of a nomogram that integrated maternal age, CL, uterine curettage, C-reactive protein, and strain of AI to predict sPTB in singleton gestations with a short CL, no prior sPTB, and treatment with vaginal progesterone. This model offered a personalized risk assessment for sPTB. As was illustrated in the Results section, a 40-year-old woman with a CL of 12 mm, C-reactive protein level of 12 mg/L, strain of AI of 0.35, GDM, absence of HDP, and a history of uterine curettage had a 93% probability of experiencing sPTB. This underscored the potential need for tailored preventive measures to mitigate the adverse outcomes of sPTB. The risk assessment could be recalculated based on varying clinical conditions, allowing for dynamic evaluation of sPTB risk. Such an approach could effectively guide patients towards individualized interventions. Validation of the nomogram model revealed its robust discrimination and calibration capabilities, with net benefits observed in both the training and validation cohorts.

This study presented 4 notable limitations. Firstly, strain elastography is a qualitative method, with its efficacy largely being contingent upon the proficiency of the sonographer. Given the potential risks associated with transmitting a focused beam in close proximity to the fetus [39,40], we opted not to incorporate shear wave elastography into our study. Secondly, due to the retrospective nature of this study, there were instances of missing data for certain participants. Thirdly, the nomogram exclusively encompassed data from women in their second trimester. The optimal gestational week for predicting sPTB risk remained undetermined. The choice of the second trimester was strategic, aligning with the customary gestational age window for performing anomaly scans, thus aiding clinical implementation. Lastly, the limited sample size underscored the need for a multi-center, large-scale, and prospective study to further corroborate these results.

## 5 Conclusions

The nomogram, which incorporated patient characteristics and cervical elastography data from the second trimester, presented a visually intuitive and dependable model for predicting sPTB in singleton gestations with a short CL, no prior sPTB, and treatment using vaginal progesterone. This method held promise as an effective tool for sPTB prediction, and its broader applicability necessitated further validation through expansive, multi-center, and prospective trials.

## Ethical approval

The ethical committee of Seventh People's Hospital of Shanghai University of Traditional Chinese Medicine approved the study (No. IEC-C-014-V1.0).

## Informed consent

Written informed consents were obtained from patients.

## Funding sources

This research received no external funding.

## Author contribution statement

Yongkang Sun; Feng Lian: Conceived and designed the experiments; Performed the experiments; Analyzed and interpreted the data; Contributed reagents, materials, analysis tools or data; Wrote the paper.

Yuanyuan Deng; Sha Liao: Performed the experiments; Analyzed and interpreted the data.

Ying Wang: Conceived and designed the experiments; Performed the experiments; Contributed reagents, materials, analysis tools or data; Wrote the paper.

## Data availability statement

Data included in article/supplementary material/referenced in article.

## Declaration of competing interest

The authors declare that they have no known competing financial interests or personal relationships that could have appeared to influence the work reported in this paper.
