# Supplementary material for: Development and validation of a nomogram to predict spontaneous preterm birth in singleton gestation with short cervix and no history of spontaneous preterm birth
Source: Heliyon. 2023 Sep 27;9(10):e20453. doi: 10.1016/j.heliyon.2023.e20453 (PMC10543363; doi:10.1016/j.heliyon.2023.e20453)
Supplement: Multimedia component 1 [file mmc1.docx]

Supplementary Table 1 Baseline characteristics of patients between the term delivery and preterm delivery group in the training cohort

| Variable | Term delivery group (n=319) | sPTB group (n=73) | *P* value |
| --- | --- | --- | --- |
| **Patient characteristics** |  |  |  |
| Maternal age, years, mean±SD | 33.07±8.46 | 37.90±8.42 | <0.001* |
| BMI before pregnancy, kg/m^2^, mean±SD | 25.28±5.86 | 24.73±5.58 | 0.464* |
| Cervical length, mm, median (IQR) | 17.20 (12.65, 21.25) | 13.18 (11.15, 15.21) | <0.001^$^ |
| Parity, n (%) |  |  | 0.047^#^ |
| 0 | 208 (65.2) | 57 (78.1) |  |
| ≥1 | 111 (34.8) | 16 (21.9) |  |
| Uterine curettage, n (%) |  |  | <0.001^#^ |
| Yes | 20 (6.3) | 20 (27.4) |  |
| No | 299 (93.7) | 53 (72.6) |  |
| Alcohol drinking history, n (%) |  |  | 0.536^#^ |
| Yes | 33 (10.3) | 10 (13.7) |  |
| No | 286 (89.7) | 63 (86.3) |  |
| Smoking history, n (%) |  |  | 0.511^#^ |
| Yes | 3 (0.9) | 2 (2.7) |  |
| No | 316 (99.1) | 71 (97.3) |  |
| Family history of diabetes mellitus, n (%) |  |  | 0.853^#^ |
| Yes | 30 (9.4) | 8 (11.0) |  |
| No | 289 (90.6) | 65 (89.0) |  |
| History of hypertension, n (%) |  |  | 0.186^#^ |
| Yes | 9 (2.8) | 5 (6.8) |  |
| No | 310 (97.2) | 68 (93.2) |  |
| GDM, n (%) |  |  | 0.001^#^ |
| Yes | 27 (8.5) | 17 (23.3) |  |
| No | 292 (91.5) | 56 (76.7) |  |
| HDP, n (%) |  |  | 0.003^#^ |
| Yes | 20 (6.3) | 13 (17.8) |  |
| No | 299 (93.7) | 60 (82.2) |  |
| Anemia, n (%) |  |  | 0.755^#^ |
| Yes | 41 (12.9) | 11 (15.1) |  |
| No | 278 (87.1) | 62 (84.9) |  |
| C-reactive protein, mg/L, median (IQR) | 7.50 (3.40, 10.90) | 9.90 (5.50, 14.20) | <0.001^$^ |
| Urinary tract infection, n (%) |  |  | 0.896^#^ |
| Yes | 26 (8.2) | 5 (6.8) |  |
| No | 293 (91.8) | 68 (93.2) |  |
| Reproductive tract infection, n (%) |  |  | 1.000^#^ |
| Yes | 45 (14.1) | 10 (13.7) |  |
| No | 274 (85.9) | 63 (86.3) |  |
| Inter-pregnancy interval, months, mean±SD | 38.49±19.24 | 43.07±28.36 | 0.097^#^ |
| Household yearly income (yuan), n (%) |  |  | 0.264^#^ |
| < 50,000 | 170 (53.3) | 33 (45.2) |  |
| ≥50,000 | 149 (46.7) | 40 (54.8) |  |
| Emergency cerclage |  |  | 0.842^#^ |
| Yes | 9 (2.8) | 3 (4.1) |  |
| No | 310 (97.2) | 70 (95.9) |  |
| Assisted reproductive technologies, n (%) |  |  | 0.429^#^ |
| Yes | 16 (5.0) | 6 (8.2) |  |
| No | 303 (95.0) | 67 (91.8) |  |
| Antibiotics use, n (%) |  |  | 0.595^#^ |
| Yes | 73 (22.9) | 14 (19.2) |  |
| No | 246 (77.1) | 59 (80.8) |  |
| **Strain in the cervix** |  |  |  |
| AI, median (IQR) | 0.25 (0.19, 0.33) | 0.34 (0.29, 0.40) | <0.001^$^ |
| PI, mean±SD | 0.31±0.12 | 0.32±0.11 | 0.411* |
| AE, mean±SD | 0.41±0.11 | 0.40±0.11 | 0.759* |
| PE, mean±SD | 0.44±0.11 | 0.43±0.12 | 0.678* |

*, for independent sample t-test; ^#^, for chi-square test; ^$^, for Mann-Whitney U-test. SD, standard deviation; BMI, body mass index; GDM, gestational diabetes mellitus; HDP, hypertensive disorders of pregnancy; AI, anterior lip of internal os; AE, anterior lip of external os; PI, posterior lip of internal os; PE, posterior lip of external os; sPTB, spontaneous preterm birth.
